# Epicardial adipose tissue and malignant ventricular arrhythmias in phospholamban p.(Arg14del) variant carriers

**DOI:** 10.1093/eurheartj/ehaf118

**Published:** 2025-03-11

**Authors:** Belend Mahmoud, Moniek G P J Cox, Remco de Brouwer, Myrthe Y C van der Heide, Thomas M Gorter, Laura M G Meems, Arthur A M Wilde, Dirk J van Veldhuisen, Rudolf A de Boer, B Daan Westenbrink

**Affiliations:** Department of Cardiology, University Medical Centre Groningen, University of Groningen, Groningen, The Netherlands; Department of Cardiology, University Medical Centre Groningen, University of Groningen, Groningen, The Netherlands; Department of Geriatrics, University Medical Centre Groningen, University of Groningen, Groningen, The Netherlands; Department of Cardiology, Amsterdam Cardiovascular Sciences, Heart Failure and Arrhythmias, Amsterdam UMC location, University of Amsterdam, Amsterdam, The Netherlands; Department of Cardiology, University Medical Centre Groningen, University of Groningen, Groningen, The Netherlands; Department of Cardiology, University Medical Centre Groningen, University of Groningen, Groningen, The Netherlands; Department of Cardiology, Amsterdam Cardiovascular Sciences, Heart Failure and Arrhythmias, Amsterdam UMC location, University of Amsterdam, Amsterdam, The Netherlands; Member of the European Reference Network for Rare, Low Prevalence and Complex Diseases of the Heart: ERN GUARD-Heart, Amsterdam, The Netherlands; Department of Cardiology, University Medical Centre Groningen, University of Groningen, Groningen, The Netherlands; Member of the European Reference Network for Rare, Low Prevalence and Complex Diseases of the Heart: ERN GUARD-Heart, Amsterdam, The Netherlands; Department of Cardiology, Erasmus Medical Centre, Cardiovascular Institute, Thorax Centre, Rotterdam, The Netherlands; Department of Cardiology, University Medical Centre Groningen, University of Groningen, Groningen, The Netherlands

**Keywords:** Epicardial adipose tissue, Ventricular arrhythmias, Phospholamban, Cardiomyopathy, Magnetic resonance imaging

## Introduction

The pathogenic p.(Arg14del) variant in the phospholamban (*PLN*) gene can cause a severe cardiomyopathy characterized by a high burden of malignant ventricular arrhythmias (MVA).^1,2^ A considerable, yet poorly understood heterogeneity in the burden of arrhythmias is observed among individuals with this variant.^3^ Epicardial adipose tissue (EAT) has recently emerged as a potential driver of arrhythmogenicity.^4^ While associations between EAT and atrial fibrillation have already been established,^5,6^ the relationship between EAT and ventricular arrhythmias remains poorly understood. We conducted a retrospective association study to assess whether ventricular EAT volume is associated with the incidence of MVA in individuals with the *PLN* p.(Arg14del) variant.

## Methods

**Figure 1:**
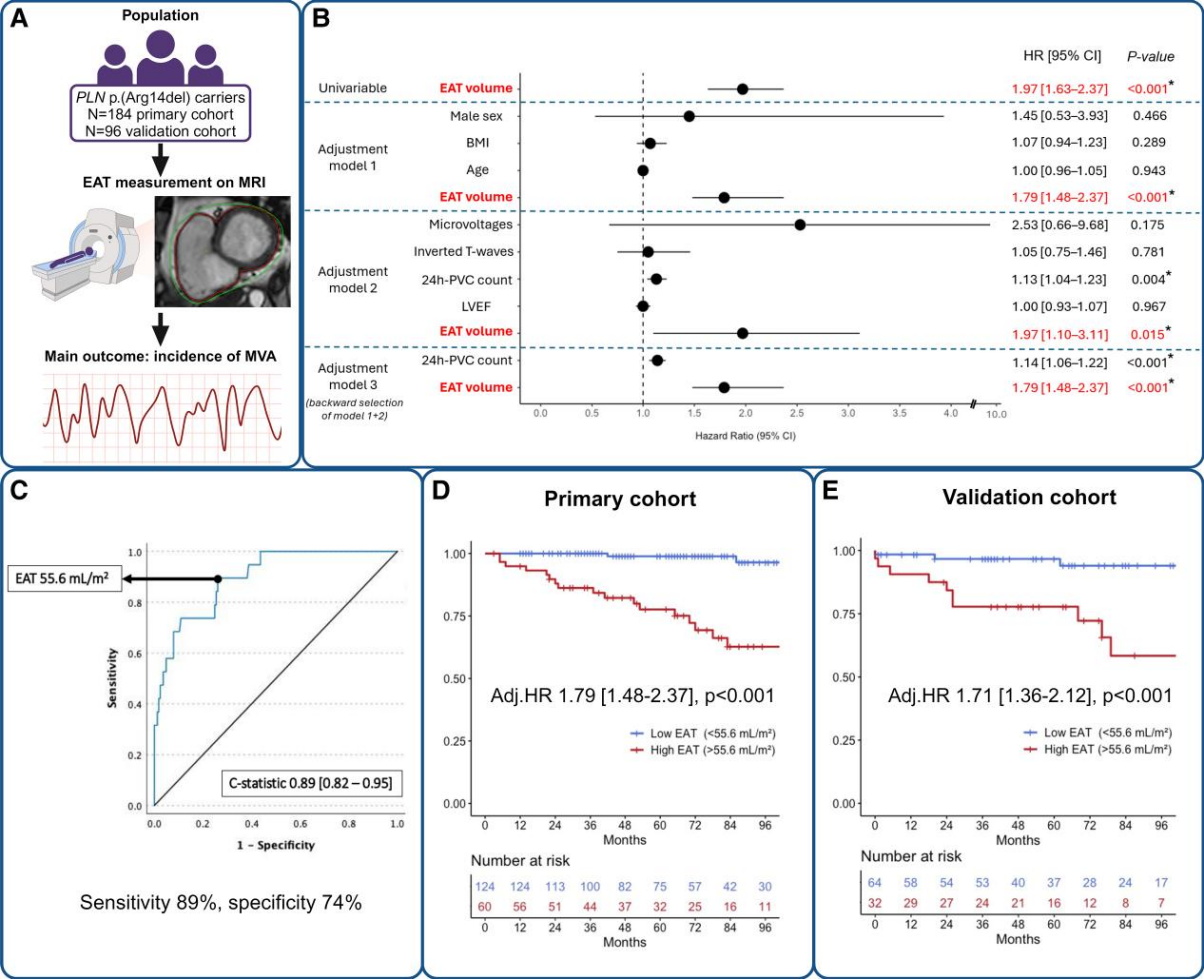
(*A*) Simplified graphical visualization of the methodology, showing population, EAT measurement on MRI, and outcome. Epicardial adipose tissue measurement is depicted on one short-axis cine stack of the heart on MRI, basal slice. Red/Bold: myocardial border. Green: visceral layer of the pericardium. The space in between represents EAT. (*B*) Forest plot showing (adjusted) HR for EAT univariably, EAT adjusted for factors influencing EAT volume (Model 1), EAT adjusted for factors in the contemporary MVA risk prediction model^8^ (Model 2), EAT adjusted for all variables in Models 1 + 2 using a backward selection process, with HR only reported for the covariates that remained significant after the backward selection (Model 3). (*C*) Receiver operating characteristic curve and Harrell’s *C*-statistic assessing the discriminative ability of EAT for MVA. The arrow indicates the optimal cut-off point based on the Youden index. (*D*) Kaplan–Meier curve showing the event-free survival of MVA in the primary cohort, stratified by EAT volume, using the optimal cut-off calculated shown in *C*. (*E*) Kaplan–Meier curve showing the event-free survival of MVA in the validation cohort, stratified by EAT volume, using the optimal cut-off calculated shown in *C*. *PLN*, phospholamban gene; EAT, epicardial adipose tissue; MRI, magnetic resonance imaging; MVA, malignant ventricular arrhythmia; BMI, body mass index; PVC, premature ventricular contraction; LVEF, left ventricular ejection fraction; HR, hazard ratio; CI, confidence interval; Adj.HR, adjusted hazard ratio

Individuals with the *PLN* p.(Arg14del) variant with a cardiac magnetic resonance imaging (MRI) scan available at the University Medical Centre Groningen were retrospectively included. Ventricular EAT volumes were measured manually using Circle Cardiovascular Imaging (Cvi42, version 5.14, Calgary, Canada) software, using a protocol described previously.^7^ The primary outcome was the incidence of MVA, defined as sustained ventricular tachycardia (VT), ventricular fibrillation (VF), or appropriate implantable cardioverter defibrillator shock intervention. Patients with a history of MVA at baseline, inadequate MRI scan quality, or without follow-up, were excluded. The primary outcome was validated in a *PLN* p.(Arg14del) cohort from the Amsterdam University Medical Centre (*Figure 1A*).

Data are presented as mean ± standard deviation, median [interquartile range], or numbers (percentage). Variables were compared using Mann–Whitney *U* test, independent *t*-test, or Fisher’s exact test where appropriate. Linear regression was used to assess correlations between EAT volume and 24 h premature ventricular contraction (PVC) count, and left and right ventricular ejection fraction (LVEF/RVEF). Multivariable Cox regression was used to assess the association between EAT volume and MVA, using several models: Model 1, variables influencing EAT volume [age, body mass index (BMI), and sex]; Model 2, variables included in the contemporary MVA risk prediction model^8^; and Model 3, backward selection of all variables used in Models 1 and 2, with a threshold of *P* < .10 for covariate elimination. Hazard ratios (HRs) are reported for the covariates that remain significant after the backward selection. In the validation cohort, EAT volume was adjusted for the covariates that remained significant in the final survival model (Model 3). Harrell’s *C*-statistic was used to assess the discriminative ability of EAT volume for the MVA outcome. In both cohorts, patients were divided into ‘low EAT’ and ‘high EAT’ groups based on the optimal EAT volume cut-off point calculated using the Youden index. Statistical analyses were performed using RStudio (version 4.1.1, Vienna, Austria), with *P* < .05 considered significant.

## Results

We included 184 patients (40 ± 15 years, 46.7% male). During 70 ± 35 months of follow-up, 19 (10.3%) patients developed MVA. Compared to those who did not develop MVA, these patients were older (48 ± 12 vs. 39 ± 15 years, *P* = .015), had higher BMI (26.9 ± 3.2 vs. 24.4 ± 3.9 kg/m^2^, *P* = .006), higher PVC count (2894 [1615–5068] vs. 70 [2–715], *P* < .001), more frequent microvoltage electrocardiograms (50% vs. 14%, *P* = .001), lower LVEF (36 [35–47] vs. 56 [52–61], *P* < .001), and higher ventricular EAT volumes (74.6 ± 18.6 vs. 49.4 ± 11.1 mL/m^2^, *P* < .001). Higher ventricular EAT volumes correlated with a higher PVC count (*R*^2^ = .229 for log_10_-PVC count, *P* < .001), lower LVEF (*R*^2^ = .427, *P* < .001), and lower RVEF (*R*^2^ = .337, *P* < .001).

Every 10 mL/m^2^ increase in ventricular EAT was associated with a 97% higher incidence of MVA (*P* < .001). This association remained significant after adjusting for age, BMI, and sex (adjusted HR [adj.HR] 1.79 [1.48–2.37], *P* < .001), the current risk prediction model^8^ (adj.HR 1.97 [1.10–3.11], *P* = .015), and all aforementioned factors in a backward selection model (adj.HR 1.79 [1.48–2.37], *P* < .001) (*Figure 1B* and *D*). Epicardial adipose tissue volume had excellent discriminative ability to predict MVA, with a *C*-statistic value of 0.89 [0.82–0.95], which was similar to the current risk prediction model. The optimal EAT volume cut-off for MVA incidence was 55.6 mL/m^2^ (sensitivity 89%, specificity 74%) (*Figure 1C*).

### External validation cohort

We included 96 patients (43 ± 15 years, 39.6% male). During 72 ± 38 months of follow-up, 15 patients (15.6%) developed MVA. These patients had higher ventricular EAT volumes than those who did not develop MVA (69.9 ± 18.6 vs. 47.7 ± 13.0 mL/m^2^, *P* < .001). In this cohort, EAT was also associated with MVA incidence after statistical adjustment (adj.HR 1.71 [1.36–2.12], *P* < .001) (*Figure 1E*).

## Discussion

In subjects with the *PLN* p.(Arg14del) pathogenic variant, ventricular EAT accumulation was associated with a higher incidence of MVA in two independent cohorts, and this association remained present after adjustments. Moreover, ventricular EAT volume demonstrated excellent discriminative ability for MVA, equivalent to that of the current risk prediction model. These findings suggest that EAT accumulation increases susceptibility to MVA.

This study is among the first and largest to associate EAT accumulation with MVA incidence. Three smaller studies have looked into this before. Sepehri Shamloo *et al*.^9^ found that EAT thickness predicted VT recurrence post-ablation. Wu *et al*.^10^ reported that higher pericardial fat volumes were linked to VT/VF occurrence in patients with heart failure. Wang *et al*.^11^ documented higher EAT volumes in patients with idiopathic VT compared to controls. Our study confirms and extends upon these findings by demonstrating this association in a large, homogeneous population with an identical genetic variant, without history of MVA, and substantial follow-up. Additionally, the multivariable adjustments and sizeable validation cohort provide a more robust link between EAT and MVA.

Our results suggest that EAT may serve as a potential substrate for ventricular arrhythmias and therefore play a role in PLN cardiomyopathy pathogenesis. Due to its high predictive value for MVA and the fact that it is a single, easy to utilize variable, there is a strong case for incorporating ventricular EAT volume into the current risk prediction model. Additionally, the association between EAT and MVA could also exist in other populations, but future studies are required to investigate this assumption.

Limitations include the retrospective nature of the study, preventing us from ascertaining causality between EAT and outcomes. Additionally, differences in characteristics between patients who did and did not develop MVA, including age and cardiac function, could not be prevented and had to be accounted for through statistical adjustments.

## Conclusion

Ventricular EAT accumulation is associated with the incidence of MVA in subjects with the *PLN* p.(Arg14del) pathogenic variant. This suggests that EAT accumulation could contribute to ventricular arrhythmogenicity.

## Declarations

### Data Availability

The data underlying this article will be shared on reasonable request to the corresponding author.

### Pre-registered Clinical Trial Number

None supplied.
